# Subcutaneous Emphysema From Bronchocavitary Subcutaneous Fistula

**DOI:** 10.4103/0970-2113.44123

**Published:** 2008

**Authors:** P. S. Shankar

**Affiliations:** MD, FAMS, FCCP DSc, Emeritus Professor of Medicine M.R. Medical College, Gulbarga J.N. Medical College, Belgaum

Extra-alveolar air in the form of subcutaneous emphysema develops in a variety of clinical settings. Escape of air into subcutaneous tissue results in subcutaneous emphysema. Simmonds in 1784 noted development of pneumomediastinum and subcutaneous emphysema following Valsalva maneuver during labour^1^.

Subcutaneous emphysema may develop spontaneously when there is a deliberate alteration in breathing pattern such as shouting, singing or even pulmonary function testing. It may also develop on straining and other involuntary alterations in breathing pattern as in child birth, violent coughing and straining at defecation. External pressure changes as in decompression (barotraumas) causing alveolar rupture, or mechanical ventilation raising intrathoracic pressure, can facilitate entry of air into the subcutaneous tissue. Subcutaneous emphysema may develop following a blunt or penetrating chest injury involving larynx, trachea or bronchi. The condition may be noted in association with pneumothorax or pneumomediastinum as a result of pathological changes in the respiratory tract. Subcutaneous emphysema may occur following chest tube insertion or tracheal intubation, or surgical procedures in upper gastrointestinal tract. Rarely it may also develop as a complication of asthma from nebulization of bronchodilators^2^. However, spontaneous subcutaneous emphysema in absence of pneumothorax or pneumomediastinum is rare.

Pandey and colleagues from Shimla, India have reported occurrence of subcutaneous emphysema in a 60-year male with tuberculous fibrocavitary disease involving right upper lobe of the lung^3^. The patient presented with sudden onset of right-sided chest pain following a bout of cough. There was swelling over upper half of the chest spreading to neck and face. Examination revealed crepitus over the chest and neck on palpation. Computerized tomography showed diffuse subcutaneous emphysema with cavity in the right upper lobe communicating with right main bronchus and subcutaneous tissue.

Bloomberg has considered the cause of non-traumatic subcutaneous emphysema to be due to weakness of either the alveolar or bronchial wall^4^. Increased intrapleural pressure following excessive and prolonged coughing, causes rupture at a weakened point allowing escape of air in the tissue. Air escapes via peribronchial or perivascular channels to the mediastinum. Then air spreads into loose alveolar tissue and gains entry to the neck. Subcutaneous emphysema in the absence of pneumomediastinum or pneumothorax is unusual. Thus the case presented by Pandey et al is unusual^3^. The pulmonary cavity must have established a communication to the subcutaneous tissue allowing seepage of air to create subcutaneous emphysema.

Air leaks develop in tuberculosis, mostly it is noted in miliary tuberculosis^5^. Das et al have encountered development of subcutaneous emphysema in Staphylococcal pneumonia, measles, Pneumocystis carinii infection, influenza pneumonia and pertusis especially among children^5^. Imaging studies help in ascertaining the cause and extent of air leak, and presence of subcutaneous gas as streaks or pockets of air. Many a times the swelling in the neck and face may be confused as angioneurotic oedema, nephrotic syndrome or superior vena cava obstruction.

The treatment is palliative. The underlying disease has to be treated and further air leak has to be prevented. The condition is self-limiting and the accumulated air gets resolved by resorption. Sometimes skin incisions are needed.
